# Long noncoding RNA dysregulation in ischemic heart failure

**DOI:** 10.1186/s12967-016-0926-5

**Published:** 2016-06-18

**Authors:** Simona Greco, Germana Zaccagnini, Alessandra Perfetti, Paola Fuschi, Rea Valaperta, Christine Voellenkle, Serenella Castelvecchio, Carlo Gaetano, Nicoletta Finato, Antonio Paolo Beltrami, Lorenzo Menicanti, Fabio Martelli

**Affiliations:** IRCCS Policlinico San Donato, Via Morandi, 30, 20097 San Donato Milanese, Milan, Italy; Goethe University, Frankfurt, Germany; Istituto di Anatomia Patologica Universitaria, Azienda Ospedaliero Universitaria “S. Maria della Misericordia”, Udine, Italy

## Abstract

**Background:**

Long noncoding RNAs (lncRNAs) are non-protein coding transcripts regulating a variety of physiological and pathological functions. However, their implication in heart failure is still largely unknown. The aim of this study is to identify and characterize lncRNAs deregulated in patients affected by ischemic heart failure.

**Methods:**

LncRNAs were profiled and validated in left ventricle biopsies of 18 patients affected by non end-stage dilated ischemic cardiomyopathy and 17 matched controls. Further validations were performed in left ventricle samples derived from explanted hearts of end-stage heart failure patients and in a mouse model of cardiac hypertrophy, obtained by transverse aortic constriction. Peripheral blood mononuclear cells of heart failure patients were also analyzed. LncRNA distribution in the heart was assessed by in situ hybridization. Function of the deregulated lncRNA was explored analyzing the expression of the neighbor mRNAs and by gene ontology analysis of the correlating coding transcripts.

**Results:**

Fourteen lncRNAs were significantly modulated in non end-stage heart failure patients, identifying a heart failure lncRNA signature. Nine of these lncRNAs (CDKN2B-AS1/ANRIL, EGOT, H19, HOTAIR, LOC285194/TUSC7, RMRP, RNY5, SOX2-OT and SRA1) were also confirmed in end-stage failing hearts. Intriguingly, among the conserved lncRNAs, *h19*, *rmrp* and *hotair* were also induced in a mouse model of heart hypertrophy. CDKN2B-AS1/ANRIL, HOTAIR and LOC285194/TUSC7 showed similar modulation in peripheral blood mononuclear cells and heart tissue, suggesting a potential role as disease biomarkers. Interestingly, RMRP displayed a ubiquitous nuclear distribution, while H19 RNA was more abundant in blood vessels and was both cytoplasmic and nuclear. Gene ontology analysis of the mRNAs displaying a significant correlation in expression with heart failure lncRNAs identified numerous pathways and functions involved in heart failure progression.

**Conclusions:**

These data strongly suggest lncRNA implication in the molecular mechanisms underpinning HF.

**Electronic supplementary material:**

The online version of this article (doi:10.1186/s12967-016-0926-5) contains supplementary material, which is available to authorized users.

## Background

It is estimated that heart failure (HF) is the cardiovascular disease with the worse rate of morbidity, mortality, accounting for one of the highest health care costs in the western world. Indeed, the 1-year-survival rate of patients with end-stage HF is about 50 % [1].

The adverse left ventricle (LV) remodeling process, leading to the clinical syndrome of HF, involves several deregulated proteins and is characterized in the adult heart by the reactivation of fetal cardiac gene expression [2]. This scenario of transcriptional control is also complicated by the addition of epigenetic mechanisms. The encyclopedia of DNA elements (ENCODE) project indicates that at least 80 % of the genome is functional and is transcribed both into protein coding RNAs (about 1.1–1.5 %) and in a much larger quantity of non-coding, regulatory RNAs, arbitrarily divided into long (lncRNAs, >200 nt), and short (<200 nt) ncRNAs [3, 4].

While dysregulation and role of short ncRNAs, in particular of miRNAs, has been extensively explored [5–7], the involvement of lncRNAs in specific physiological and pathological processes [5, 8], as well as in cardiovascular diseases [9–16], is still in its early stages of study.

In this study, in order to investigate the molecular mechanisms underpinning HF during its progression, we profiled the expression of 83 lncRNAs, known to be implicated in human diseases, in LV biopsies of non end-stage HF patients.

## Methods

### Patient selection and tissue collection

The investigation was conducted in conformity with the principles outlined in the Helsinki Declaration and with the Italian laws and guidelines, and was authorized by local Ethics Committee (protocol #2438, 27/01/2009). All specimens were taken after informed consent disclosing future use for research. Left ventricle (LV) cardiac biopsies from patients affected by non end-stage dilated hypokinetic ischemic cardiomyopathy where collected during surgical ventricular restoration procedure performed as described previously [17, 18]. For each patient, two biopsies were collected from the non-ischemic and dysfunctional remote myocardium: one was immediately immersed in RNAlater (Qiagen GmbH) and stored at 4 °C for <24 h before RNA extraction, and the other one was fixed in 10 % neutral buffered formalin (NBF) and paraffin embedded for RNA in situ hybridization and immunohistochemistry assays. HF patients’ characteristics are described in Table 1. Peripheral blood mononuclear cells (PBMC) were isolated from the peripheral blood of HF patients the day before surgery or from healthy controls by Histopaque Ficoll (Sigma Diagnostics, St. Louis, MO, USA) gradient centrifugation as described previously [19].

**Table 1:** Clinical characteristics of the study population

Characteristics	HF patients
Age (years) (median ± SE)	65.0 ± 0.6
Sex (male/female)	17/1
Diabetics (%)	28
BSA (m^2^) (median ± SE)	1.8 ± 0.02
GFR (ml/min) (median ± SE)	64.2 ± 1.6
Glucose (mg/dl) (median ± SE)	105 ± 1.5
Time from MI (months) (median ± SE)	5.5 ± 10.6
NYHA class (%)	1 = 5; 2 = 55; 3 = 39; 4 = 1
DD (mm) (median ± SE)	62.5 ± 0.5
SD (mm) (median ± SE)	52.0 ± 0.5
LVEF (%) (median ± SE)	26.5 ± 0.4
EDV (ml) (median ± SE)	216.5 ± 2.7
ESV (ml) (median ± SE)	151.0 ± 2.2
E/A (median ± SE)	1.25 ± 0.1
*Medications (%)*	
Oral antidiabetic agents	15
Insulin	20
Statins	75
ACEIs	70
β-Blockers	80
Aspirin	95
Loop diuretics	85
Nitrates	35

ACEI angiotensin converting enzymes inhibitor, BSA body surface area, DD diastolic diameter, SD systolic diameter, E/A diastolic function ratio, EDV end-diastolic volume, ESV end-systolic volume, GFR glomerular filtration rate, LVEF left ventricular ejection fraction, MI myocardial infarction, NYHA New York Heart Association Functional Classification

We have also analyzed 11 post-ischemic end-stage heart failure patients [10 males and 1 female, aged 59 ± 2.4 years (median ± SE)]. Among these, 8 LV samples were collected by an expert pathologist from explanted hearts of patients that underwent cardiac transplantation; the remaining 3 end-stage samples were collected during the implantation of LV assist device (LVAD). Care was taken to avoid necrotic or fibrotic areas. Samples of ~2 mm^3^ were snap frozen in liquid nitrogen and kept at −80 °C until they were analyzed. The end-stage HF patients were classified as NYHA class 3 (82 %) and class 4 (18 %), showed a LV transverse diameter of 130 ± 5.3 mm (median ± SE), measured by the pathologist after explantation, and ejection fraction of 22 ± 2.9 % (median ± SE). Sample harvesting was conducted after approval of the Ethics Committee of Udine (2 August 2011, reference number 47,831), in accordance with the Declaration of Helsinki, once written informed consent was obtained from each enrolled patient.

The control group was composed by age- and sex-matched subjects, who had died for causes different from stroke, ischemia, or cachexia for chronic diseases (n = 17, females/males = 6/10; 58.3 ± 3.4 years old). Left ventricle samples were excised and processed with less than 30 min cold ischemic time and snap frozen (XpressBANK, Asterand Biosciences).

### Mouse transverse aortic constriction (TAC)

All experimental procedures complied with the Guidelines of the Italian National Institutes of Health and with the Guide for the Care and Use of Laboratory Animals (Institute of Laboratory Animal Resources, National Academy of Sciences, Bethesda, MD, USA) and were approved by the institutional Animal Care and Use Committee (IACUC n. 709). TAC was performed in 2 months old C57BL/6 J male mice with a 27-gauge needle [20]. Before all surgical procedures, mice were anesthetized with an intraperitoneal injection of 10 mg/kg Xilazine (Intervet Farmaceutici, Italy) and 100 mg/kg Ketamine (Ketavet 100; Intervet Farmaceutici, Italy). After the surgery, mice were allowed to recover at 37 °C. Sham operated mice received the same procedure except for the ligation of the aorta. Only mice showing a pressure gradient >60 mmHg measured by Doppler echocardiography 7 days after TAC were included in the analysis. Sham-operated mice were used as controls [21].

### Formalin-fixed and paraffin-embedded (FFPE) genomic DNA isolation and single nucleotide polymorphism (SNP) analysis

Genomic DNA was isolated from twenty 10 μm-thick paraffin-embedded tissue sections. Following deparaffinization twice for 5 min in xylene, DNA was extracted using QIAamp DNA FFPE Tissue kit (Qiagen, USA) following manufacturer’s instructions. Four CDKN2-AS SNPs (RS1333040, RS1333049, RS10757278 and RS2383207) were assessed by TaqMan SNP Genotyping Assay on ABI 7900 real time PCR platform (Life Technologies, Thermo Fisher Scientific Inc., MA, USA). 20 ng of DNA were amplified in a reaction volume of 25 μl, containing 12.5 μl of 2× TaqMan Master Mix, 1.25 μl of 20× Assay working stock solution and 6.25 μl of nuclease-free water. Real time PCR conditions were: one cycle of 2 min at 50 °C, one cycle of 10 min at 95 °C; 40 cycles of 15 s at 95 °C and 1 min at 60 °C.

### RNA isolation, lncRNA profiling and RT-qPCR

Total RNA from tissues was extracted using TRIzol (Life Technologies, Thermo Fisher Scientific Inc., MA, USA) as described previously [17, 22]. Sizing, quantitation and quality of the extracted RNAs was checked by Nanodrop ND-1000 (Nanodrop, Thermo Fisher Scientific Inc., MA, USA) and Bioanalyzer 2100 (Agilent Technologies). Long noncoding RNA expression profiles were measured using the disease-related human lncRNA Profiler (SBI, System Biosciences) (Additional file 1: Table S1). One µg of total RNA was retro-transcribed using the SuperScript III Reverse Transcriptase kit (Life Technologies, Thermo Fisher Scientific Inc., MA, USA) according to the manufacturer’s instructions. cDNAs were analyzed using the SYBR-GREEN qPCR method (Life Technologies, Thermo Fisher Scientific Inc., USA) according to the manufacturer’s instructions. Data are deposited in Gene Expression Omnibus repository (GEO GSE77399 http://www.ncbi.nlm.nih.gov/geo/query/acc.cgi?acc=GSE77399). After median Ct value normalization, relative RNA expression was calculated with the 2^−ΔΔCt^ method [23]. Significantly (p < 0.05) modulated lncRNAs with a Ct ≤ 33 in at least one group and displaying a 2^−ΔΔCt^ fold change ≥|1| were used for further validation. Validation was carried out with newly designed specific primers by SYBR-GREEN qPCR (Additional file 1: Table S2), using UBC for normalization.

### RNA in situ hybridization assay and immunohistochemistry

In situ hybridization was performed using the QuantiGene ViewRNA system (Affymetrix) according to the manufacturer’s instructions. Briefly, 3-μm-thick sections were derived from FFPE-embedded biopsies and mounted on Superfrost Plus Gold glass slides (Thermo Fisher Scientific). Next, deparaffinized sections were hybridized with oligonucleotide probes conjugated with alkaline phosphatase (LP-AP) type 1, followed by staining with fast blue substrate, counterstaining with Hoechst 3332 (Sigma-Aldrich Co.) and mounted with VectaMount AQ medium (Vector Lab., USA).

To identify endothelial cells, serial LV sections were deparaffinized, microwave-treated and incubated with anti-human CD31 mouse monoclonal antibody (M0823, Dako). After incubation with biotinylated secondary antibodies and with ABC complex (Vectastain), the reaction was revealed with diaminobenzidine (DAB, vector) and counterstained with Hematoxylin.

For both in situ hybridization and immunohistochemistry, at least two blinded readers carried out the analysis and random images were acquired using an Axio Imager M.1 microscope equipped with an Axiocam MRc5 camera (Zeiss) and AxioVision software (Zeiss).

### Transcriptomic analysis

To analyze the relationship between lncRNAs and mRNAs differentially expressed in the same HF samples, we used the dataset GEO GSE26887 [17]. We identified the mRNA transcripts significantly correlated to the differentially expressed lncRNAs by using Co-LncRNA platform [24] and Spearman’s rank correlation test, considering p ≤ 0.05 as statistical significance threshold. The lists of the correlated mRNAs were used to predict the enriched pathways by using WebGestalt [25].

For neighboring gene analysis, the 300 kb of genomic sequence upstream and downstream the HF modulated lncRNAs were identified by using UCSC Genome Browser (GRCh38/hg38 assembly) [26]. Significant differential expression of neighboring genes lying in these regions was determined according to GSE26887 dataset [17].

For the comparison of transcriptomic changes in LV and PBMCs of HF patients, the following GEO datasets were analyzed: for LV, GSE26887 [17], and for PBMCs, GSE9128 [27] and GSE1869 [28]. Enriched pathways were obtained from each list of significantly (p ≤ 0.01) differentially expressed mRNAs and common deregulated pathways were plotted as a Venn’s diagram using BioVenn web application [29].

### Statistics

Continuous variables are expressed as mean ± standard error. Gaussian distribution was tested by using the Kolmogorov–Smirnov test. For group-wise comparisons, Mann–Whitney test (two groups) or *t* test (two groups) were used as appropriate. All tests were performed 2-sided and a p ≤ 0.05 was considered as statistically significant. For statistical analysis, GraphPad Prism v.4.03 software (GraphPad Software Inc.) was used. For CDKN2B-AS SNPs analysis, observed allele frequencies were compared to reported allele frequencies in HapMap–CEU European using an exact multinomial test using R package EMT [30].

## Results

### Identification of lncRNAs deregulated in HF patients

In order to identify lncRNAs deregulated in HF, LV biopsies of 13 HF patients and 12 age-and sex-matched controls were analyzed. Worth noting is that myocardial biopsies were harvested from the non-ischemic portion of the LV (remote zone) in non end-stage HF patients, allowing to investigate the molecular mechanisms underpinning the disease during its progression. As expected, histological analysis revealed cardiomyocyte hypertrophy (Additional file 2: Figure S1B), in keeping with decreased alpha-myosin heavy chain (MYH6) and increased Natriuretic Peptide A (NPPA) mRNA levels (Additional file 2: Figure S1C, D).

**Fig. 1:**
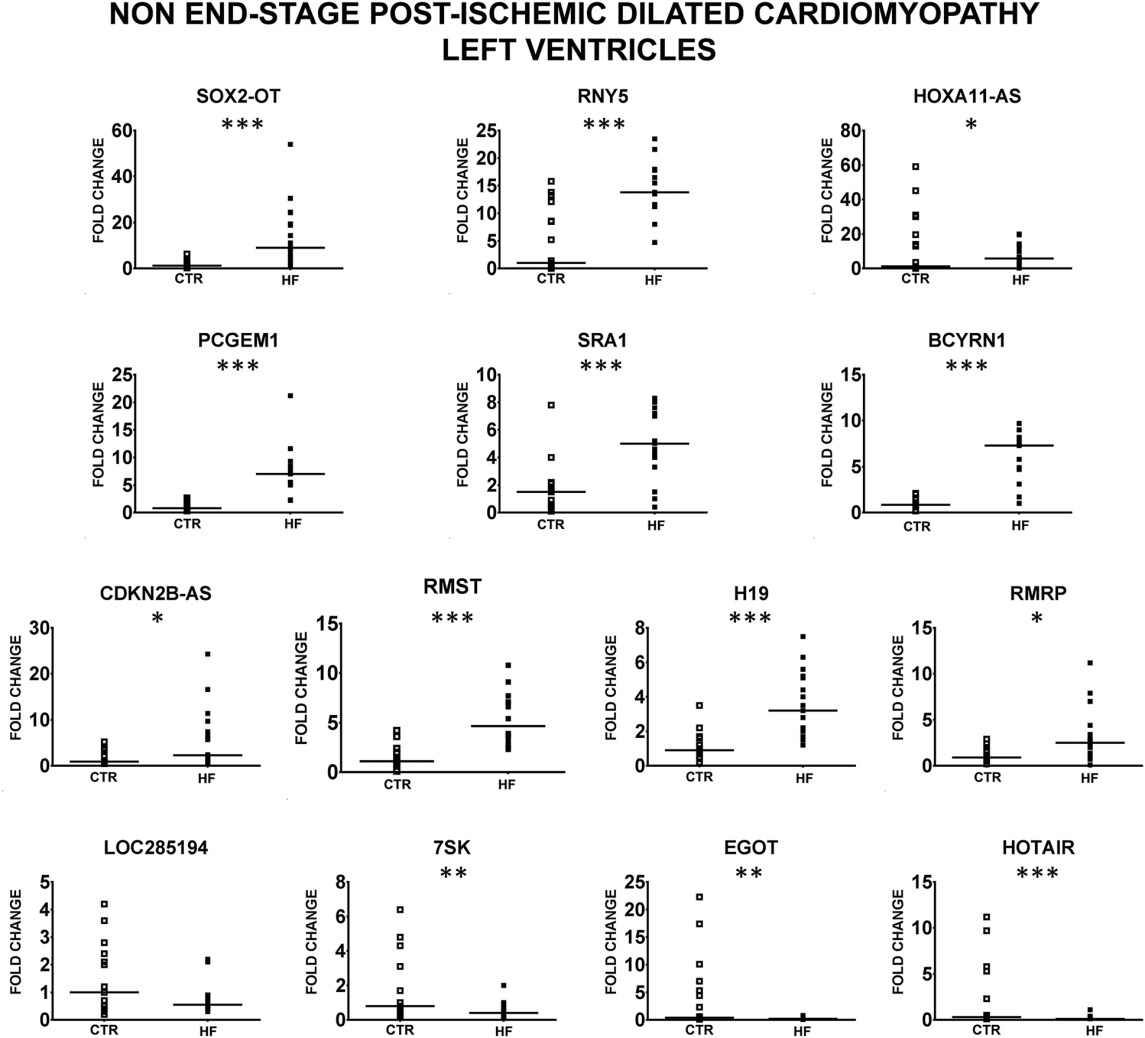
Deregulated lncRNAs in non end-stage HF patients. LncRNAs identified by profiling as deregulated in HF were validated by RT-qPCR in LV biopsies of 18 non end-stage ischemic dilated cardiomyopathy (HF) and 17 control subjects (CTR). *Dot plots* indicate fold change values of each subject with respect to controls. The *horizontal bars* indicate median values (*p ≤ 0.05, **p ≤ 0.01; ***p ≤ 0.001)

Total RNA was extracted from LV biopsies of HF patients and controls, checked for quality (Additional file 2: Figure S2) and the gene expression levels of 83 disease-related lncRNAs (Additional file 1: Table S1), were measured by RT-qPCR. LncRNAs profiling showed that 53 and 33 lncRNAs were detectable (Ct ≤ 33) in HF patients and controls, respectively. We found that 27 lncRNAs were significantly modulated (20 up- and 7 down) at least onefold in HF compared with CTR subjects (Additional file 2: Figure S3A). Since the profiling results relied on commercial primers with undisclosed sequences, after designing new couples of unique primers, interrogating the Reference Sequence [31] of each lncRNA (Additional file 1: Table S2), significantly modulated lncRNAs were validated by RT-qPCR assays in more patients and controls (18 and 17, respectively). We found that 13 lncRNAs (10 up- and 3 downregulated) were significantly modulated in HF patients compared with CTR subjects, identifying a HF lncRNA signature (Fig. 1 and Additional file 2: Figure S3B). LOC285194 (also known as TUSC7) was down-modulated, but exhibited only borderline significance (p < 0.055). One additional limitation applies to LOC285194, as well as to EGOT, since the primer couples interrogating the relevant reference sequences did not interrogate certain shorter isoforms (Additional file 1: Table S2).

Unfortunately, the number of patients analyzed precluded further stratification or a correlation analysis with clinical parameters.

CDKN2B-AS gene (also known as ANRIL) is located in a region with several SNPs that correlate to increased genetic susceptibility to coronary artery diseases and type 2 diabetes [32, 33]. In genomic DNA extracted from 26 HF patients FFPE sections, we analyzed the expression of four CDKN2B-AS SNPs (RS1333040, RS1333049, RS10757278 and RS2383207) already known to be associated with cardiovascular diseases [34, 35]. No statistically significant difference between observed and HapMap allele frequencies (Additional file 1: Table S3) or correlation with RNA expression levels (data not shown) were found, possibly due to the low number of subjects analyzed.

### Validation in chronic end-stage HF patients

In order to validate the identified HF lncRNA signature, we analyzed 11 RNAs derived from left ventricle samples of patients with chronic ischemic end-stage HF. These patients exhibited more severe clinical conditions and LV dilation compared to the patients used for lncRNA profiling.

**Fig. 2:**
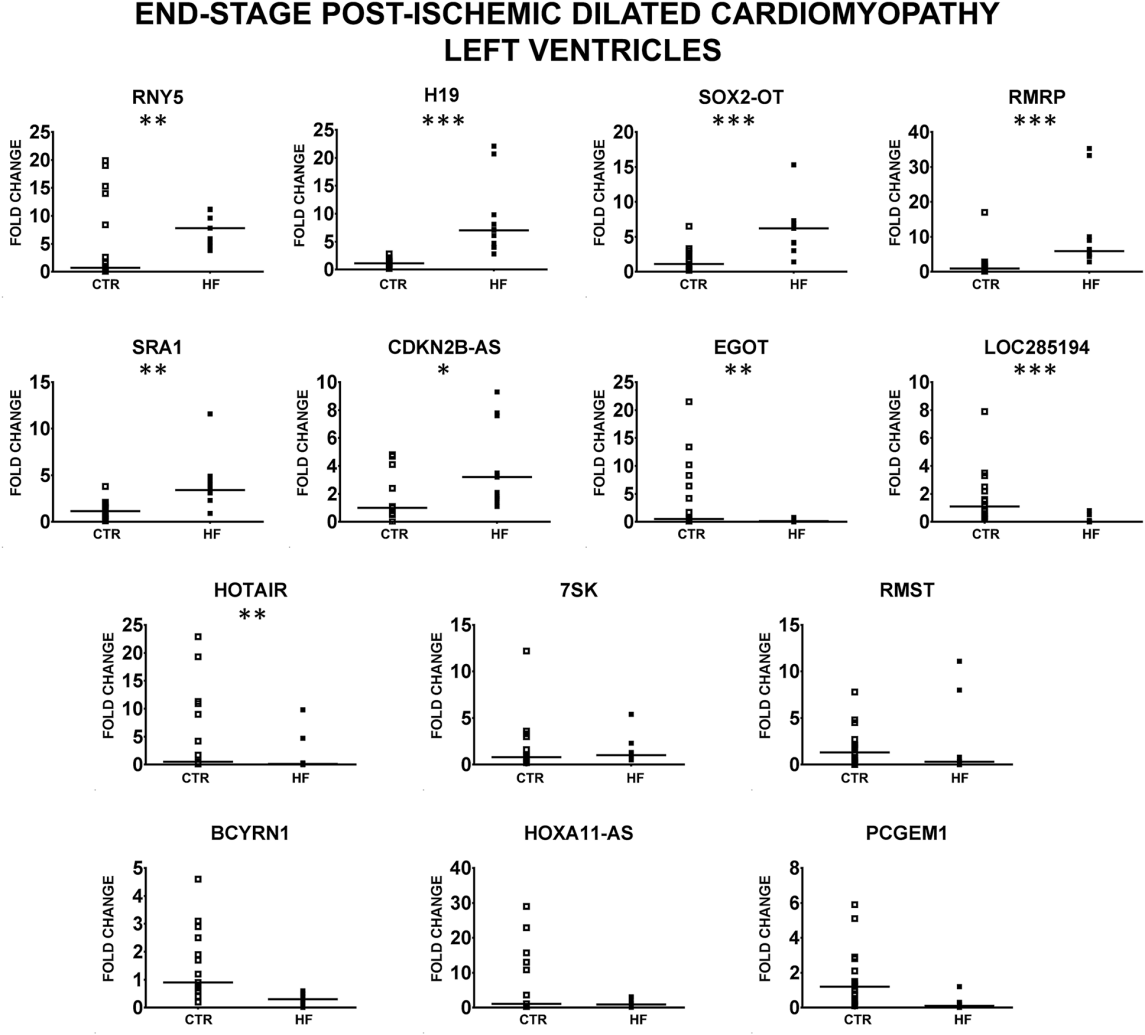
Validation of HF lncRNAs in end-stage HF patients. HF lncRNAs were validated by RT-qPCR in LV samples of 11 end-stage HF patients and 17 control subjects. *Dot plots* indicate fold change values of each subject with respect to controls. The *horizontal bars* indicate median values (*p ≤ 0.05, **p ≤ 0.01; ***p ≤ 0.001)

We found that 9 lncRNAs (CDKN2B-AS1, EGOT, H19, HOTAIR, LOC285194, RMRP, RNY5, SOX2-OT and SRA1) were significantly modulated in a concordant manner in both end- and non end-stage HF patients (Fig. 2; Additional file 1: Table S5). HOXA11-AS was similarly modulated as well, but failed to reach statistical significance. Conversely, the expression of LOC285194 displaying a borderline significant decrease in non end-stage patients was strongly and very significantly (p < 0.001) inhibited in end-stage patients. Finally, RMST expression level was increased in non end-stage HF patients and significantly decreased in end-stage HF patients, underlining the differences between the two patient groups.

### HF lncRNAs modulation in a mouse model of cardiac hypertrophy

**Fig. 3:**
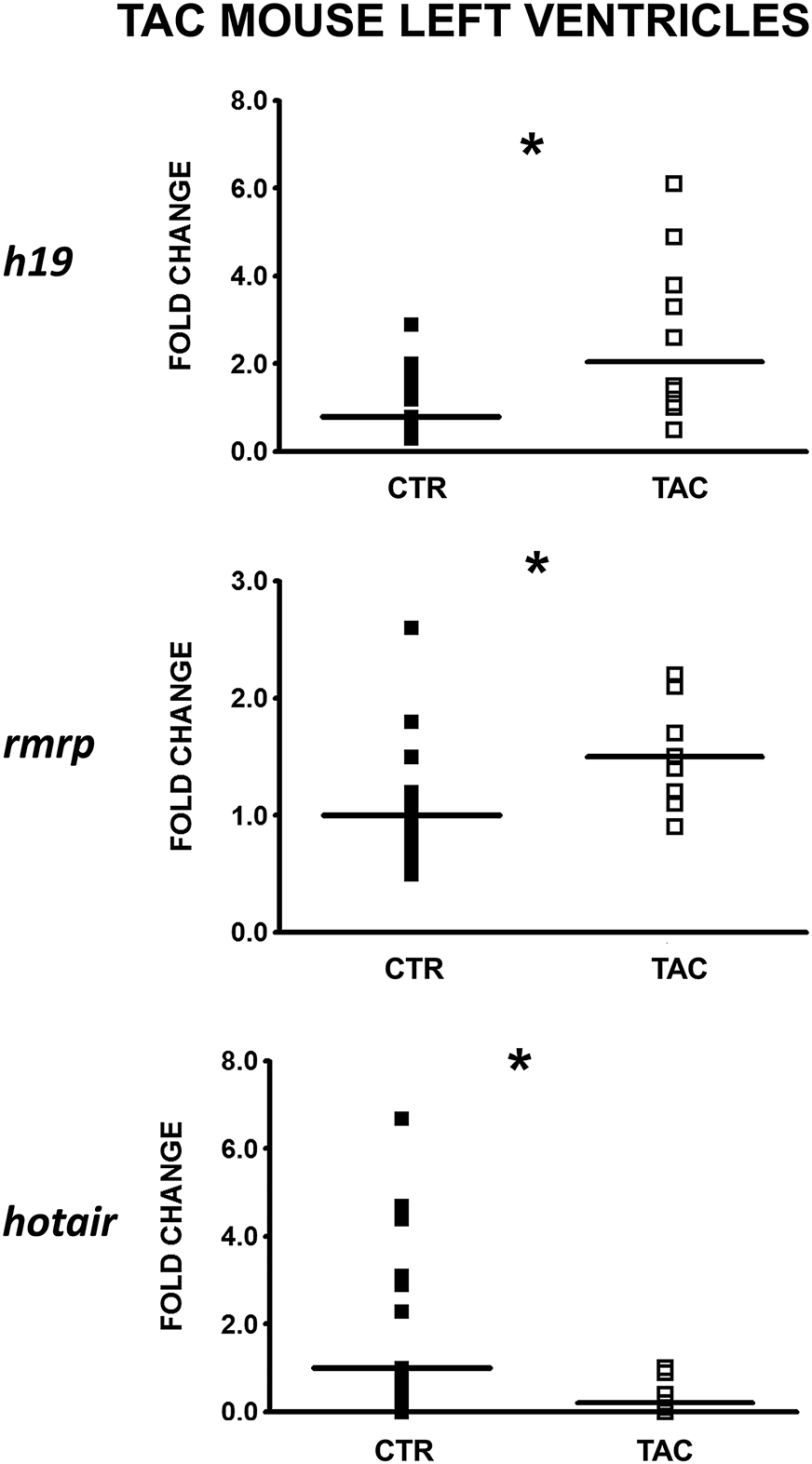
Validation of HF lncRNAs in a mouse model of cardiac hypertrophy. LV pressure overload was induced by TAC and *H19*, *rmrp* and *hotair* RNA levels were measured by RT-qPCR 7 days after surgery. *Dot plots* indicate fold change values of each subject with respect to controls. The *horizontal bars* indicate median values (TAC = 10; CTR = 15; *p ≤ 0.05)

Cardiomyocyte hypertrophy is a hallmark of post-ischemic HF patients (Additional file 2: Figure S1). Thus, we used a mouse model of cardiac hypertrophy obtained by transverse aortic constriction (TAC) to assay the expression of the identified lncRNAs. Gene expression was analyzed at day 7 after TAC, in order to examine early responses to hypertrophy, avoiding other potentially confounding effects. As expected [36, 37], SERCA2 (*atp2a2*) transcript levels decreased, while natriuretic peptides A and B (*nppa* and *nppb*) as well as Actin α1 (*acta*) transcripts increased in TAC hearts compared to controls (Additional file 2: Figure S4). When the expression of mouse-conserved lncRNAs (H19, HOXA11-AS, RMST, RMRP, SOX2OT and HOTAIR, Additional file 2: Figure S5) was assayed, we found that *h19*, *rmrp* and *hotair* were significantly modulated in a manner similar to the human counterpart (Fig. 3).

### HF lncRNAs expression in the peripheral blood

Peripheral blood mononuclear cells (PBMCs) are a particularly attractive biomarker source because of the accessibility of peripheral blood and its straightforward preparation. Furthermore, inflammation and the underlying cellular and molecular mechanisms seem to play a crucial pathological role in the progression toward HF [38, 39]. Accordingly, gene ontology analysis of the transcriptomic alterations observed in the LV and PBMCs of HF patients showed that the majority of the dysregulated pathways and functions were in common between the two tissues (Additional file 1: Table S8; Additional file 2: Figure S7).

**Fig. 4:**
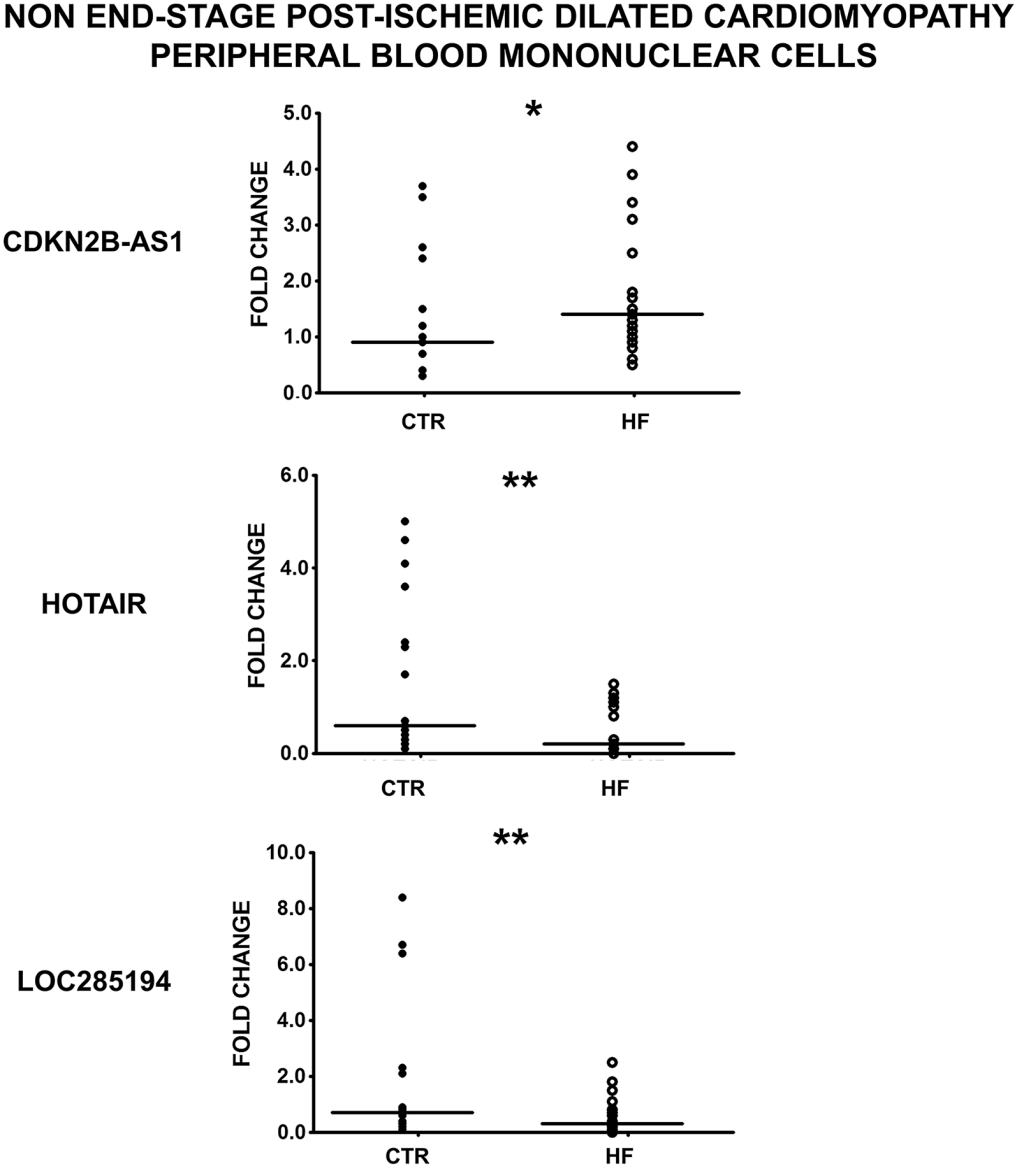
Validation of HF lncRNAs in PBMCs from HF patients and controls. HF lncRNA levels were measured in PBMCs derived from 25 non end-stage ischemic dilated cardiomyopathy (HF) patients and 18 age-and sex-matched controls. Dot-plots show CDKN2B-AS1, HOTAIR and LOC285194 levels in each patient measured by RT-qPCR; the lines indicate the median values (*p ≤ 0.05, **p ≤ 0.01)

Thus, we measured the expression of the HF deregulated lncRNAs in PBMCs from 25 HF patients and 18 healthy individuals by RT-qPCR. Interestingly, we found that CDKN2B-AS1, HOTAIR and LOC285194 showed similar modulation in PBMCs and heart tissue, suggesting a potential as disease biomarkers (Fig. 4).

### Cell localization of HF lncRNAs

**Fig. 5:**
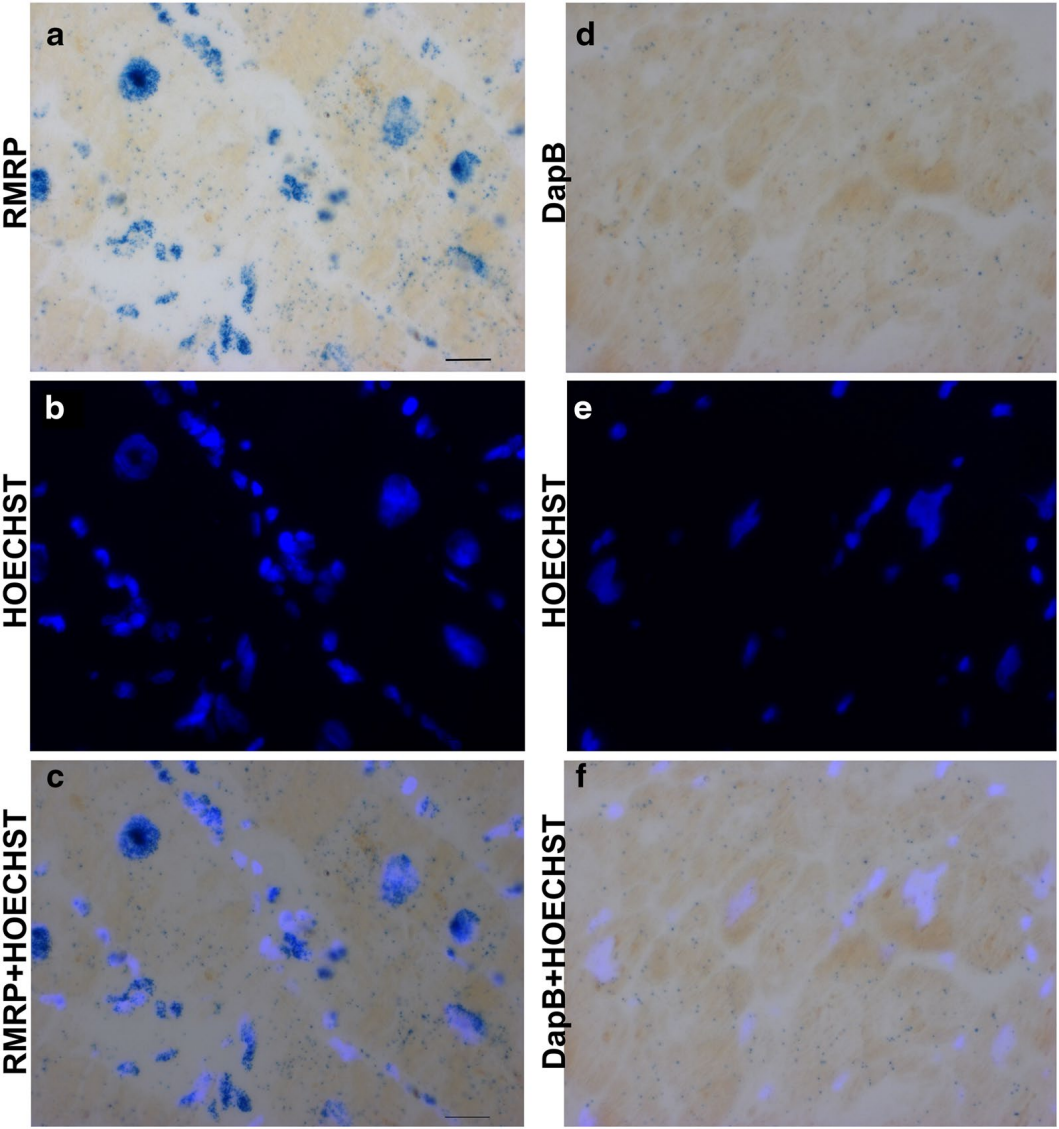
Localization of RMRP RNA in failing hearts. Sections were derived from FFPE LV biopsies of HF patients and RMRP RNA was detected by in situ hybridization in *blue*, using bright field microscopy (**a**, **c**). As negative control, hybridization was also carried out with a probe for an exogenous RNA, *DapB* of *B. subtilis* (**d**, **f**). Nuclei were detected by HOECHST 3332 counterstaining using fluorescence microscopy (**b**, **e**). The merge of RMRP and HOECHST 3332 signals **c** shows that RMRP RNA has a ubiquitous, mostly nuclear distribution. Representative pictures are shown (n = 10; *calibration* *bar* = 20 µm; magnification 40×)

In situ hybridization assay can provide important information about the cellular and sub-cellular distribution of lncRNAs. However, this assay is technically challenging, especially for low expressed transcripts [40, 41]. We managed to detect RMRP, one of the most abundant among the HF lncRNAs, according to the Ct values in RT-qPCR (19.8 ± 0.3). Indeed, while absolute Ct values are not quantitative, low Ct levels generally correspond to high expression levels [42]. Figure 5 shows that RMRP displayed a ubiquitous distribution and nuclear intracellular localization.

**Fig. 6:**
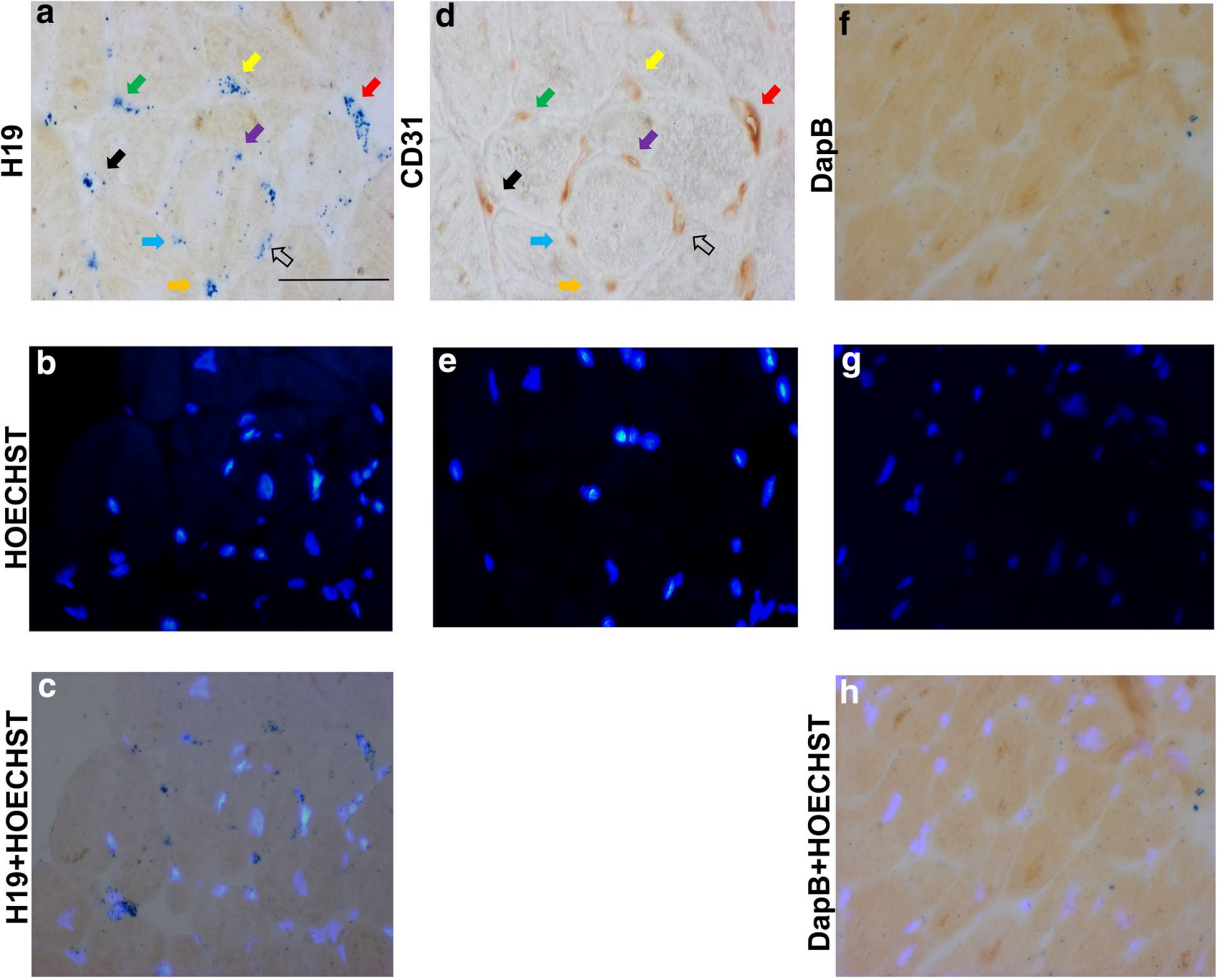
Localization of H19 RNA in HF myocardium. Sections were derived from FFPE LV biopsies of HF patients and H19 RNA was detected by in situ hybridization in blue, using bright field microscopy (**a**, **c**). Vascular structures were identified in serial sections by staining for CD31, an endothelial marker (**d**). *Colored arrows* indicate matching H19/CD31 signals. For negative control, in situ hybridization was also carried out with a probe for an exogenous RNA, *DapB* of *B. subtilis* (**f**, **h**). Nuclei were detected by HOECHST 3332 counterstaining using fluorescence microscopy (**b**, **e**, **g**). The merge of H19 and HOECHST 3332 signals (**c**) shows both nuclear and cytoplasmic distribution of H19. Representative pictures are shown (n = 16; *calibration bar* = 20 µm; magnification 40×)

Global H19 levels in the heart did not seem particularly high (25.8 ± 0.3). However, we were able to detect H19 in left ventricle sections, possibly due to its localized accumulation, mainly interstitial; indeed, the staining of serial sections with H19 and anti-CD31 antibody indicated a likely vascular localization (Fig. 6). Accordingly, H19 was readily detectable by RT-qPCR in cultured endothelial cells [43]. In keeping with results obtained in other tissues [44, 45], H19 RNA staining was both cytoplasmic and nuclear.

The specificity of H19 and RMRP hybridization signals was confirmed by negative controls performed using a probe for an exogenous gene, *DapB* of *Bacillus subtilis* (Fig. 5d, f and Fig. 6f, h).

### Interactions between coding and noncoding transcriptomic changes

In order to gain insight into the role played by the HF lncRNAs, we took advantage of a previous transcriptomic analysis performed on the same RNAs using microarray (GEO GSE26887) [17].

**Fig. 7:**
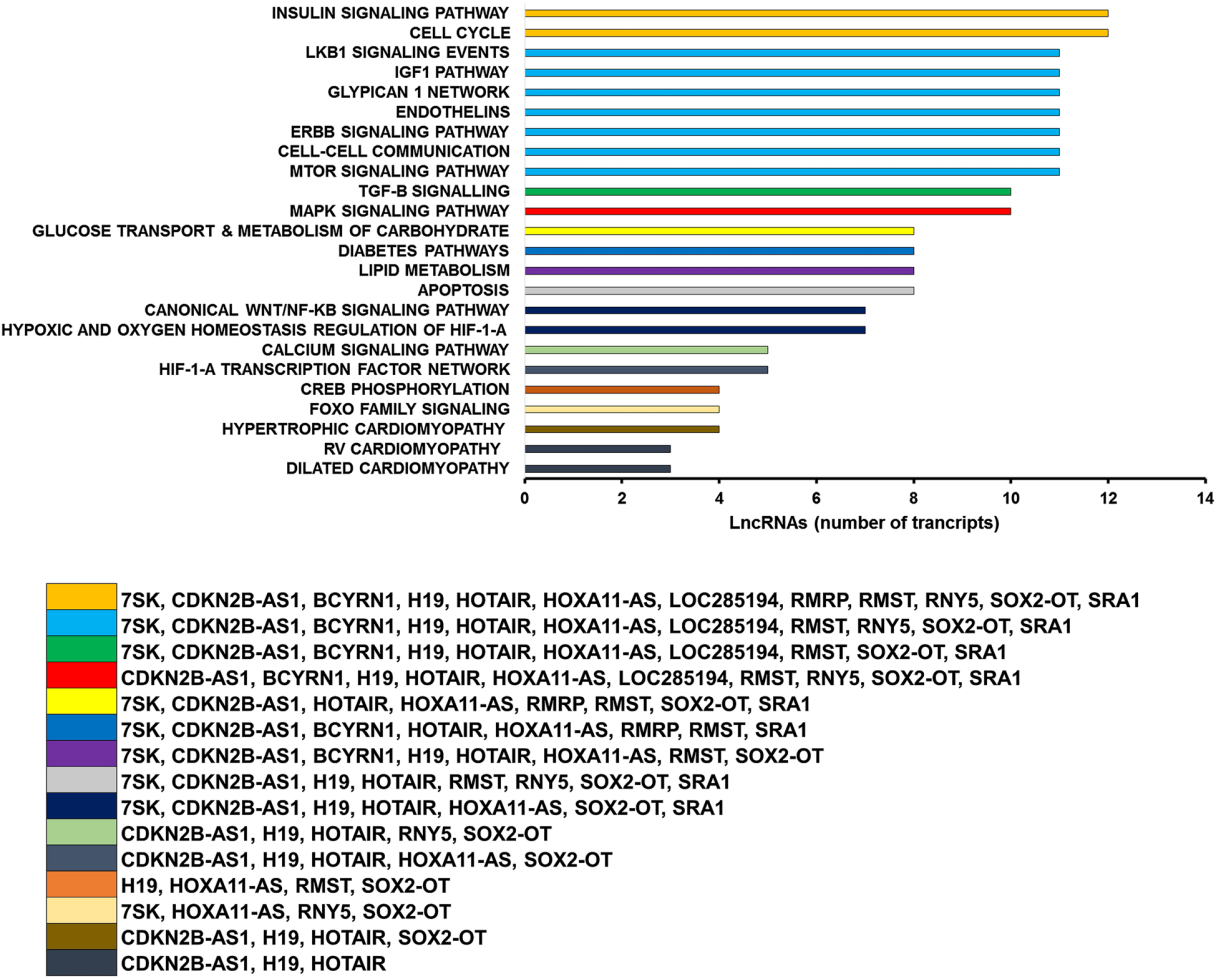
Pathways enrichment analysis of mRNAs correlating with HF lncRNAs. mRNAs transcripts (GEO GDS4314) and lncRNAs differentially expressed in HF were used for correlation analysis. The transcripts correlating with HF lncRNA were analyzed using WebGestalt software for pathways enrichment, and enriched pathways in common in ≥3 HF lncRNAs and relevant for cardiovascular physiopathology are shown. Pathways and functions deriving by the same HF lncRNA combination are indicated with the same *color*

mRNAs and HF lncRNAs were used for correlation analysis, which identified 10,257 and 8852 transcripts displaying significant direct or inverse correlation, respectively, with at least one HF lncRNA (Additional file 1: Table S6; Additional file 2: Figure S5). Next, we used the list of mRNAs correlating to each HF lncRNA for gene ontology analysis, and then selected those enriched pathways found in at least three HF lncRNAs. Indeed, we reasoned that different HF lncRNAs might have an additive effect in modulating specific pathways. We identified several important pathways for cardiovascular disease, such as insulin, IGF1 and glucose signaling pathways, TGF-β pathway, as well as hypoxic and oxygen homeostasis regulation of HIF-1-α (Fig. 7, and for a complete list, Additional file 1: Table S7).

LncRNAs can act in *cis* to regulate neighboring genes [46]. Thus we studied the expression of coding genes located 300 kb upstream or downstream the deregulated lncRNAs in non end-stage HF LV samples. To this aim, we analyzed the differentially expressed mRNAs in GSE26887 dataset [17] and identified 19 lncRNA/mRNA couples potentially involved in HF disease mechanisms (Additional file 1: Table S4).

Albeit correlative, these analyses strongly suggest lncRNA involvement in the molecular mechanisms underpinning HF.

## Discussion

Several lncRNAs have been shown to be involved in specific physiological and pathological processes [5, 8], as well as in cardiovascular diseases [9–16]. We identified 14 lncRNAs deregulated in non end-stage HF patients. The validity of these findings was confirmed by the fact that nine of these lncRNAs displayed a concordant deregulation in an independent group of end-stage HF patients. Interestingly, LOC285194 that was weakly modulated in non end-stage patients was strongly down modulated in explanted failing hearts, and RMST displayed an inverse modulation in end-stage and non end-stage patients. Little is known about LOC285194 and RMST functions, but it is possible to speculate that differential regulation in the two HF groups might be linked to differences in disease progression. LOC285194 was previously shown to suppress tumor cell growth [47] and is the antisense transcript of LSAMP that has been reported to be a tumor suppressor gene [48]. Interestingly, LSAMP down-regulation in coronary artery diseases is associated to atherosclerosis burden [48]. RMST physically interacts with the transcription factor SOX2 and together regulate a large pool of downstream genes implicated in neurogenesis [49].

It is well known that, while miRNAs seem to be highly conserved, longer transcripts are under diverse levels of evolutionary constraints in mammalian. As a matter of fact, some lncRNAs show a high level of nucleotide sequence conservation (>60 %) in mammalian [50], and others do not display extensive evolutionary conservation [51], precluding them from being studied using mouse models. Nevertheless, in order to investigate the potential involvement of the identified lncRNAs in one important aspect of HF, we used a mouse model of cardiac hypertrophy. In spite of the multiple differences, we found that RMRP and H19 levels increased while HOTAIR levels decreased in both human failing and mouse hypertrophic hearts, thus opening the way for further functional studies.

Also interesting is the deregulation of CDKN2B-AS1, HOTAIR and LOC285194 in PBMCs derived from HF patients. Given the importance of inflammation in HF progression [39], it is tempting to speculate that these lncRNAs might respond to inflammatory stimuli in both myocardium and PBMCs. Moreover, it is also possible that these concomitant regulations are, at least in part, due to lncRNA transfer from one cell type to another via exosomes or other vesicles [52, 53].

Since peripheral blood can be obtained with minimally invasive procedures, CDKN2B-AS1, HOTAIR and LOC285194 might have a potential as circulating biomarkers of HF. Further studies in larger patients groups are necessary to assess their validity as disease biomarkers.

Pathway enrichment analysis highlighted several important cardiovascular categories enriched in HF lncRNAs-correlated mRNAs. Although correlative [54], this analysis fits well with previously published functional studies, suggesting that the identified HF lncRNAs might have a role in HF progression and prompting validation with more direct approaches. Among the significantly downregulated lncRNAs, HOTAIR (HOX antisense intergenic RNA) is transcribed from the antisense strand of homeobox C gene locus, and, in coordination with chromatin modifying enzymes, regulates gene silencing [55, 56]. Down-regulation of HOTAIR transcript has been found in aortic valve cells exposed to cyclic stretch, a modulation mediated through WNT/β-CATENIN pathway [57]. Accordingly, we have observed the decrease of HOTAIR in a mouse model of cardiac hypertrophy due to pressure overload, indicating a possible involvement of HOTAIR in the left ventricle remodeling associated to HF. Moreover, both WNT and cardiac hypertrophy pathways were enriched categories in the gene ontologies analysis of mRNAs correlated to HOTAIR. Equally interesting is another downregulated lncRNA, the cyclin kinase Cdk9 inhibitor 7SK, which has been recently demonstrated to be involved in cardiac hypertrophy [58]. Accordingly, histological and biomarkers analysis showed cardiac hypertrophy in the non end-stage HF patients studied. Unfortunately, lack of conservation between humans and mice precluded the analysis of 7SK expression in TAC mice.

CDKN2B-AS1 is the antisense gene of CDKN2B (or p15^ink4a^), which is located in the 50 kb chromosomal locus 9p21 [59]. CDKN2B-AS1 represses the transcription of the genes in the INK4 locus by direct binding to the INK4b transcript and by recruiting the polycomb repressor complex (PRC) [60, 61].

CDKN2B-AS1 is the strongest genetic marker of human atherosclerosis and is generally considered the ‘golden standard’ for any genome wide association study of atherosclerosis-related traits [32, 33]. We analyzed the expression of four well-known cardiovascular diseases-associated variants, but we were not able to find any frequency association with the HF status, probably due to the limited number of patients analyzed.

We found that CDKN2B-AS1 RNA levels increased in HF patients compared to controls, but we did not find any correlation with the analyzed SNPs. Indeed, the association of CDKN2B-AS1 RNA expression levels and 9p21.3 risk alleles is still controversial [62]. Nonetheless, CDKN2B-AS1 RNA expression has been shown to stimulate cell proliferation, adhesion, and to reduce apoptosis, providing a potential atherosclerosis disease mechanism [63]. Noteworthily, we found that CDKN2B-AS1 RNA was also upregulated in PBMCs of HF patients, further implicating CDKN2B-AS1 in HF pathogenic mechanisms.

The steroid receptor RNA activator 1 (SRA1) gene generates both steroid receptor RNA activator protein (SRAP) as well as several non-coding SRA transcripts, depending on alternative transcription start site usage and alternative splicing [64, 65]. Friedrichs et al. [66] identified a gene cluster including SRA1 on a 600-kb linkage disequilibrium block on chromosome 5q31. 2–3 associated with human dilated cardiomyopathy in three independent Caucasian populations. Moreover, a role in heart development has been observed in zebrafish [66]. Accordingly, knock-down of SRA1 impairs cardiac function in zebrafish [66]. SRA1 is also known to stimulate cell proliferation as well as apoptosis in vivo [67], suggesting that SRA1 may be involved in HF pathogenesis.

H19 is a developmentally regulated gene with putative tumor suppressor activity [68]. Here we showed the increase of H19 levels in HF patients, both end-stage and non, and confirmed its upregulation in a mouse model of cardiac hypertrophy [69]. H19 is expressed during development of rat aorta, decreases in adult, and, interestingly, increases after vascular injury both in vivo and in vitro [70], as well as upon hypoxic stimulus [43, 71, 72]. Moreover, hyperhomocysteinemia, an independent risk factor for coronary artery diseases (CAD), increases the expression of H19 in aorta and vascular smooth muscle cells [73, 74], indicating that upregulated H19 may participate in the progression of CAD, the most common cause of HF. Recently, it has also been shown that polymorphisms in H19 are correlated with CAD [75] and CAD risk factors, such as obesity, high birth weight, and hypertension [76, 77]. In this respect, the mainly vascular localization of H19 in the heart appears particularly significant.

Among the protein coding genes neighbor to the lncRNAs, it is interesting the concordant modulation of HOTAIR and SMUG1, which is related to free-radicals response [78]. Additionally, 7SK is inversely modulated compared to GSTA4 and 5, that both are involved in gluthatione metabolism [79]. Moreover, we observed that Cathepsin D was modulated concordantly to its neighbor lncRNA H19. Cathepsin D is an autophagy-related enzyme and it has been found up-regulated in a hamster model of dilated cardiomyopathy [80].

## Conclusions

We identified 14 lncRNAs that are dysregulated in non end-stage HF patients. Validation in other patient groups and in animal experimental models corroborated the findings, and the deregulation of some of them in the peripheral blood suggests a potential as disease biomarkers. While future investigations of the identified HF lncRNA actions and dysfunctions are required, analysis of the correlated genes indicates their implication in the molecular mechanisms underpinning HF progression.

## Additional files

10.1186/s12967-016-0926-5 Disease association of the lncRNAs of the SBI "Disease-related human lncRNA profiler" plate. **Table S2: **Sequences and gene alignment of the primers designed for validation. **Table S3:** Single nucleotide polymorphism (SNPs) analysis of CDKN2-AS1. **Table S4:** Differentially expressed protein coding genes neighboring the HF modulated lncRNAs. **Table S5:** Non end-stage HF LncRNAs signature in end-stage HF patients. **Table S6:** HF-modulated mRNAs correlating with HF-modulated lncRNAs. **Table S7:** Pathway enrichment analysis of HF-modulated mRNAs correlating with HF-modulated lncRNAs. **Table S8: **Significantly enriched pathways in common between LV and PBMC datasets in HF patients.
10.1186/s12967-016-0926-5Cardiac hypertrophy of non end-stage patients. Sections were derived from FFPE LV biopsies of controls (A) and HF patients (B). Hematoxylin and eosin staining showed a clear cardiomyocyte hypertrophy. Representative pictures are shown (HF n=16; CTR n=4; calibration bar=100 µm; magnification 20X). The bar graphs show the RT-qPCR measurement of MYH6 and NPPA hypertrophy markers, that are down- and up-regulated as expected (HF=10; CTR=5; **p≤0.01; *** p≤0.001) (C and D). **Figure S2:** Quality control of RNA extracted from heart biopsies of patients and controls. Total RNA was extracted from LV samples derived from non end-stage HF (n=18, panel A), end-stage HF (n=11, panel B) or controls (n=17, panel C). Integrity and amount of RNAs were measured by Bioanalyzer electrophoresis. Representative patterns and RNA Integrity Numbers (RIN) are shown. **Figure S3:** LncRNAs profiling in non end-stage HF patients. (A) Profiling of lncRNAs by RT-qPCR in 13 HF patients and 12 age-and sex-matched controls. (B) Validation of significantly deregulated lncRNAs in 18 HF and 17 controls. The bar graph (A) and table (B) shows the average fold change values with respect to controls (*p≤0.05, **p≤0.01; *** p≤0.001). **Figure S4:** Transverse aortic constriction induces cardiac hypertrophy. LV pressure overload was induced by TAC in C57BL/6J mice and cardiac hypertrophy markers were measured by RT-qPCR, 7 days after surgery. As expected atp2a2 was down- modulated and nppa, nppb and acta were increased (TAC=10; CTR=8; *p≤0.05, **p≤0.01). **Figure S5:** Mouse-human genomic alignment of HF lncRNAs. Gene locations from GRCh38.p5 (GCA_000001405.20) and from GRCm38.p4 (GCA_000001635.6) human and mouse genome assemblies, respectively, were used to compare the lncRNA sequence alignment in human and mouse by using Clustal Omega (http://www.ebi.ac.uk/Tools/msa/clustalo/). The percentage of identity is indicated. **Figure S6:** LncRNA/mRNA correlation analysis in HF. HF lncRNAs levels correlated to the levels of the mRNA expressed in the same samples. The bar graph indicates the number of transcripts significantly correlated for each lncRNA, either positively (white) or negatively (black). **Figure S7:** Venn’s diagram of enriched pathways in common between LV and PBMCs in HF patients GSE26887, GSE9128 and GSE1869 GEO datasets. The comparison of the 89, 99 and 53 enriched pathways of GSE26887, GSE9128 and GSE1869 datasets, respectively, identified 43 enriched pathways in common. Transcriptomic changes observed in LV and PBMCs of HF patients were determined in the following GEO datasets: for LV, GSE26887, and for PBMCs, GSE9128 and GSE1869. Common deregulated pathways were plotted as a Venn’s diagram.

## Abbreviations

HF: heart failure
LV: left ventricle
lncRNA: long noncoding RNA
PBMC: peripheral blood mononuclear cells
TAC: mouse transverse aortic constriction
FFPE: formalin-fixed and paraffin-embedded
SNP: single nucleotide polymorphism
CAD: coronary artery diseases
