# Supplementary figures and images for: Long noncoding RNA dysregulation in ischemic heart failure
Source: J Transl Med. 2016 Jun 18;14:183. doi: 10.1186/s12967-016-0926-5 (PMC4912721; doi:10.1186/s12967-016-0926-5)

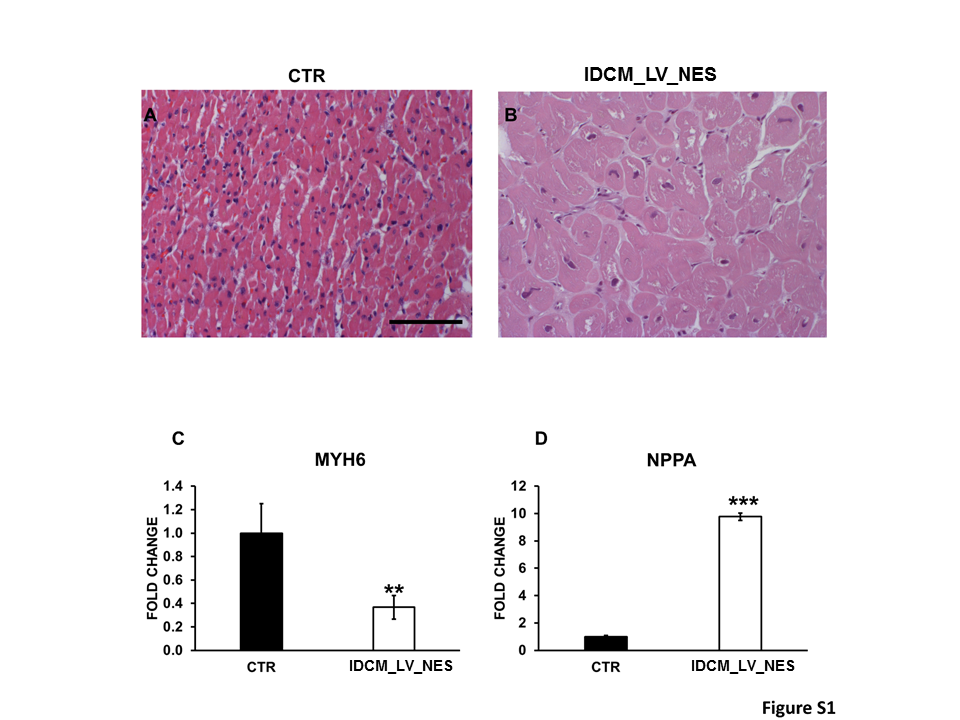


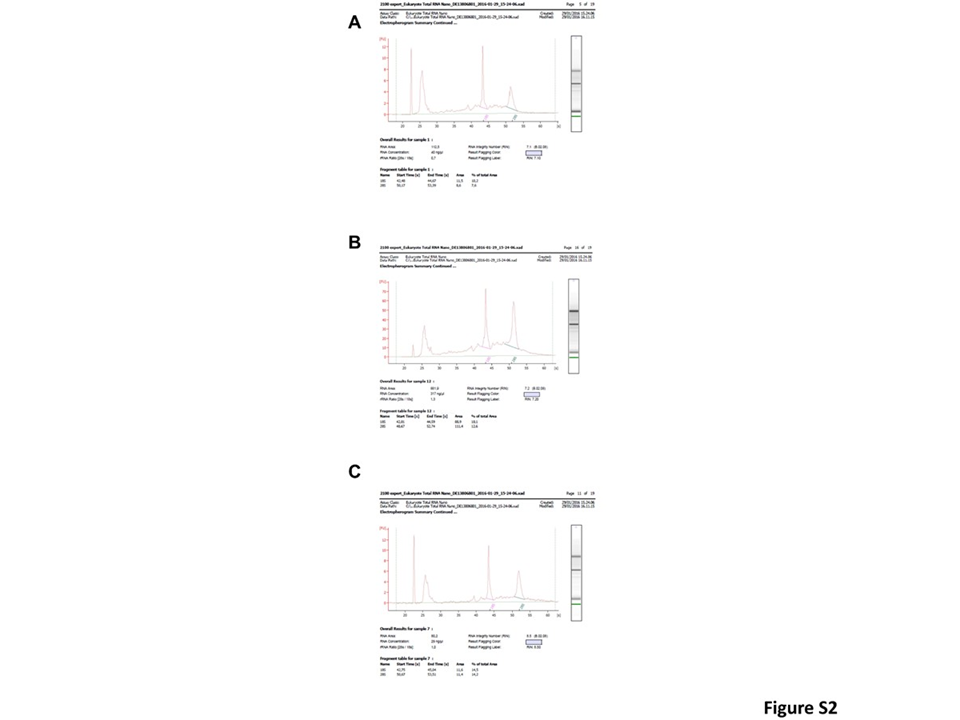


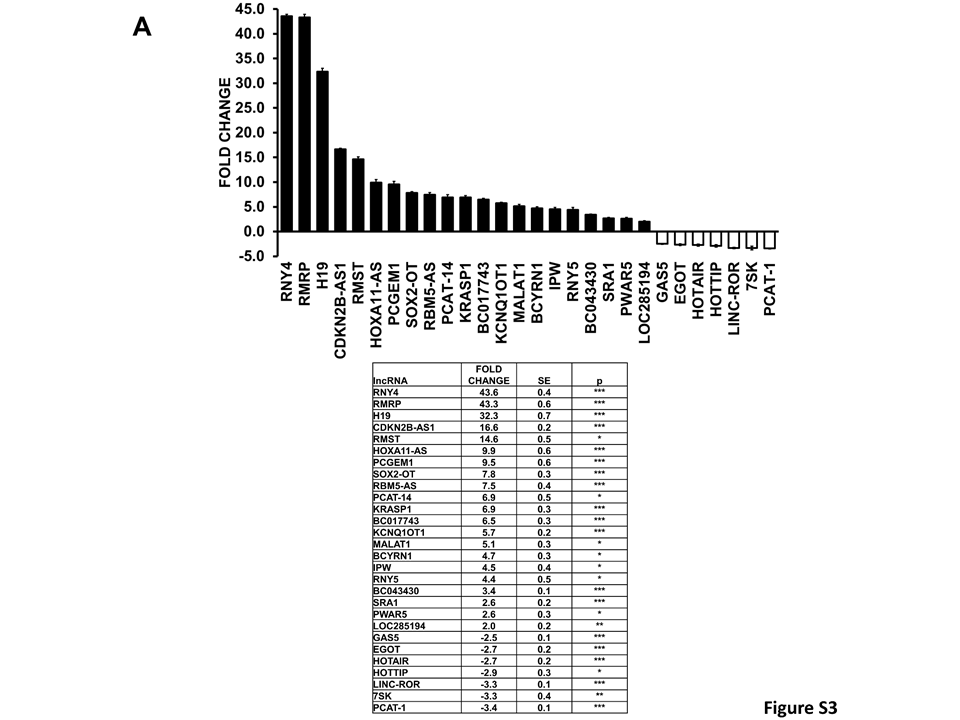


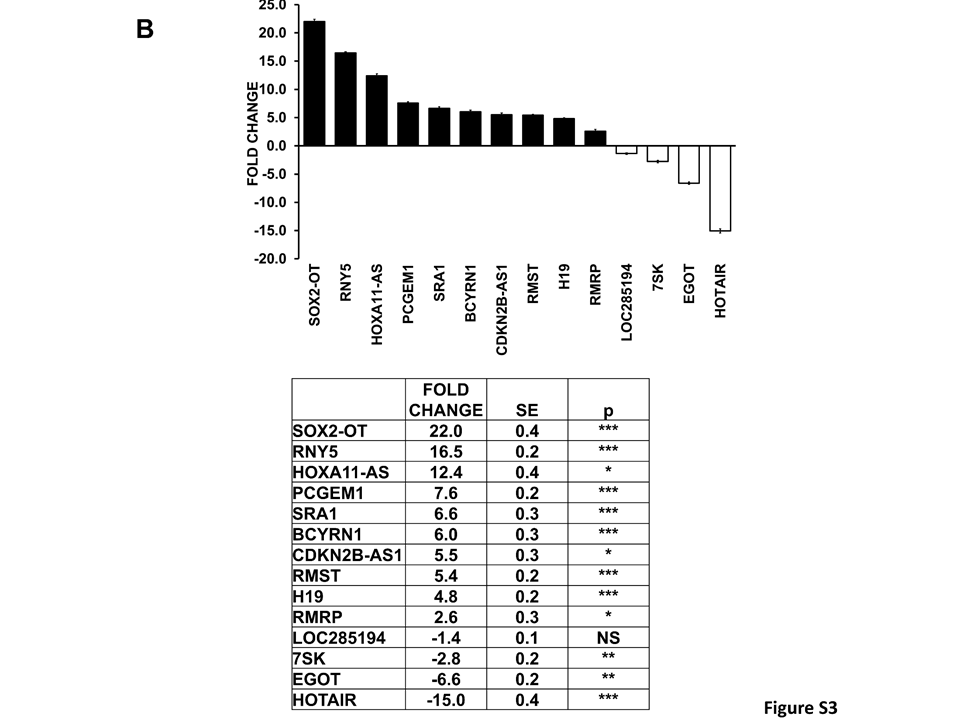


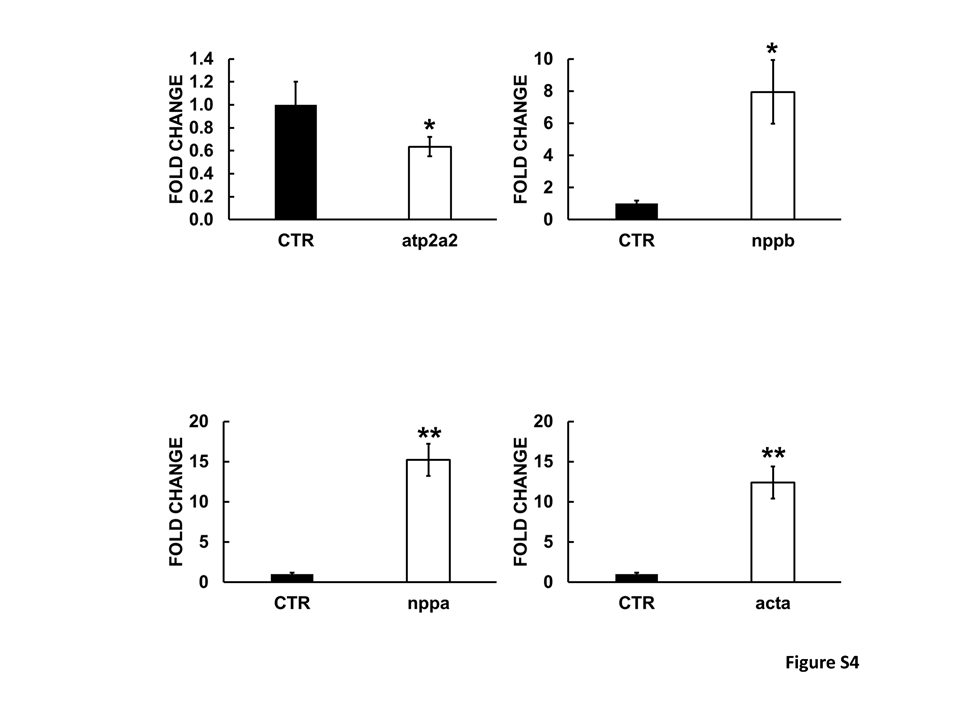


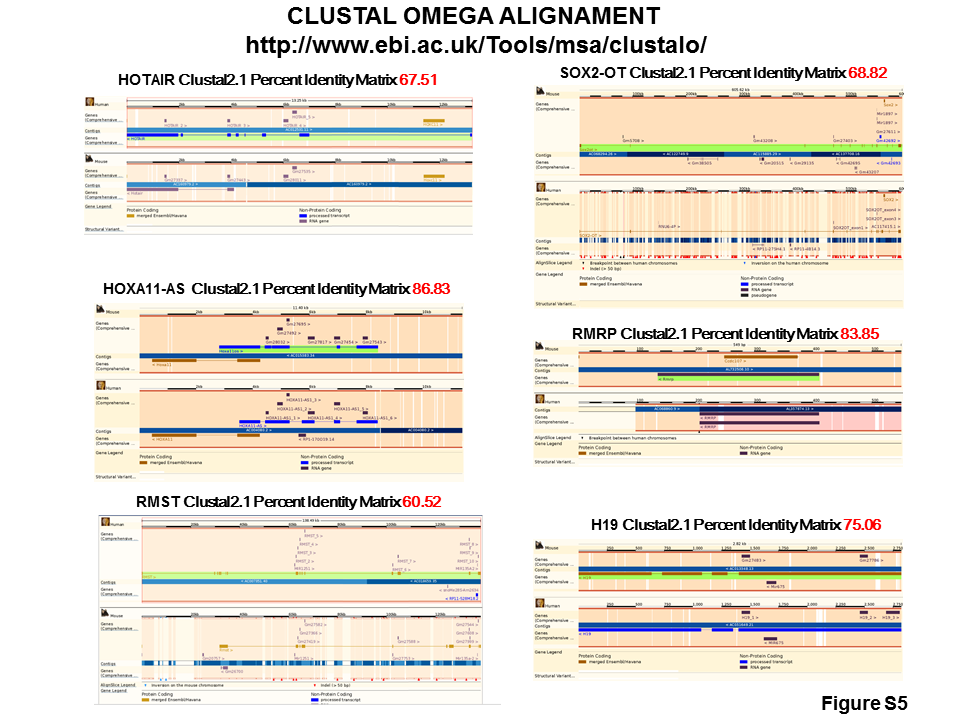


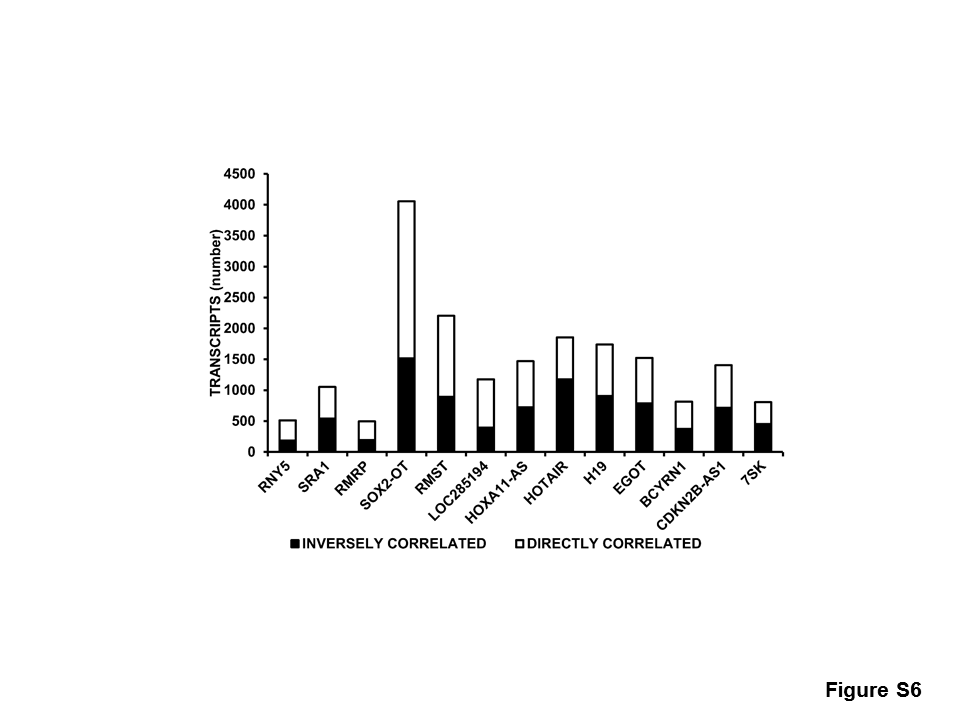


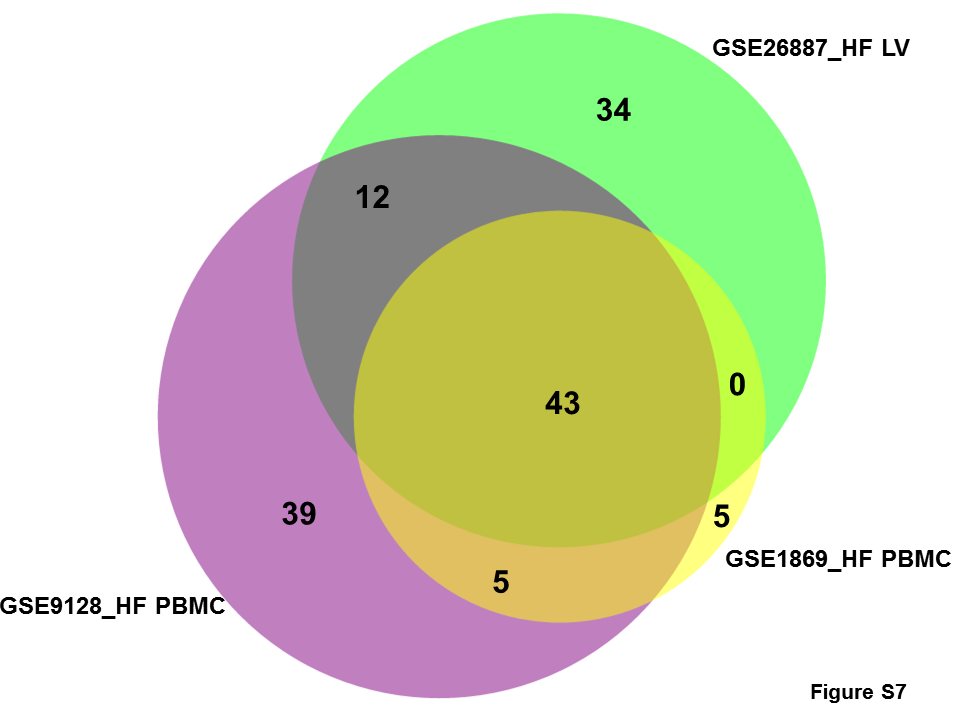

Supplement: Supplementary file 2 — 10.1186/s12967-016-0926-5Cardiac hypertrophy of non end-stage patients. Sections were derived from FFPE LV biopsies of controls (A) and HF patients (B). Hematoxylin and eosin staining showed a clear cardiomyocyte hypertrophy. Representative pictures are shown (HF n=16; CTR n=4; calibration bar=100 µm; magnification 20X). The bar graphs show the RT-qPCR measurement of MYH6 and NPPA hypertrophy markers, that are down- and up-regulated as expected (HF=10; CTR=5; **p≤0.01; *** p≤0.001) (C and D). Figure S2: Quality control of RNA extracted from heart biopsies of patients and controls. Total RNA was extracted from LV samples derived from non end-stage HF (n=18, panel A), end-stage HF (n=11, panel B) or controls (n=17, panel C). Integrity and amount of RNAs were measured by Bioanalyzer electrophoresis. Representative patterns and RNA Integrity Numbers (RIN) are shown. Figure S3: LncRNAs profiling in non end-stage HF patients. (A) Profiling of lncRNAs by RT-qPCR in 13 HF patients and 12 age-and sex-matched controls. (B) Validation of significantly deregulated lncRNAs in 18 HF and 17 controls. The bar graph (A) and table (B) shows the average fold change values with respect to controls (*p≤0.05, **p≤0.01; *** p≤0.001). Figure S4: Transverse aortic constriction induces cardiac hypertrophy. LV pressure overload was induced by TAC in C57BL/6J mice and cardiac hypertrophy markers were measured by RT-qPCR, 7 days after surgery. As expected atp2a2 was down- modulated and nppa, nppb and acta were increased (TAC=10; CTR=8; *p≤0.05, **p≤0.01). Figure S5: Mouse-human genomic alignment of HF lncRNAs. Gene locations from GRCh38.p5 (GCA_000001405.20) and from GRCm38.p4 (GCA_000001635.6) human and mouse genome assemblies, respectively, were used to compare the lncRNA sequence alignment in human and mouse by using Clustal Omega (http://www.ebi.ac.uk/Tools/msa/clustalo/). The percentage of identity is indicated. Figure S6: LncRNA/mRNA correlation analysis in HF. HF lncRNAs levels correlate [file 12967_2016_926_MOESM2_ESM.docx]
